# Optimizing Trilobatin Production via Screening and Modification of Glycosyltransferases

**DOI:** 10.3390/molecules29030643

**Published:** 2024-01-30

**Authors:** Yue Yang, Yuhan Cheng, Tao Bai, Shimeng Liu, Qiuhui Du, Wenhao Xia, Yi Liu, Xiao Wang, Xianqing Chen

**Affiliations:** 1 Jiaxing Synbiolab Biotechnology Co., Ltd., Jiaxing 314006, China; yangyue@synbiolab.cn (Y.Y.); baitao@synbiolab.cn (T.B.); liushimeng@synbiolab.cn (S.L.); duqiuhui@synbiolab.cn (Q.D.); 2 School of Ecology and Environment, Northwestern Polytechnical University, Xi’an 710072, China; ligoin@icloud.com (Y.C.); xiawenhao@synbiolab.cn (W.X.); 61liuyi@mail.nwpu.edu.cn (Y.L.)

**Keywords:** trilobatin, phloretin, glycosyltransferase, gene mining, modification

## Abstract

Trilobatin (TBL) is a key sweet compound from the traditional Chinese sweet tea plant (*Rubus suavissimus S. Lee*). Because of its intense sweetness, superior taste profile, and minimal caloric value, it serves as an exemplary natural dihydrochalcone sweetener. It also has various health benefits, including anti-inflammatory and glucose-lowering effects. It is primarily produced through botanical extraction, which impedes its scalability and cost-effectiveness. In a novel biotechnological approach, phloretin is used as a precursor that is transformed into TBL by the glycosyltransferase enzyme ph-4′-OGT. However, this enzyme’s low catalytic efficiency and by-product formation limit the large-scale synthesis of TBL. In our study, the enzyme *Md*ph-4′-OGT was used to screen 17 sequences across species for TBL synthesis, of which seven exhibited catalytic activity. Notably, PT577 exhibited an unparalleled 97.3% conversion yield within 3 h. We then optimized the reaction conditions of PT577, attaining a peak TBL bioproduction of 163.3 mg/L. By employing virtual screening, we identified 25 mutation sites for PT577, thereby creating mutant strains that reduced by-products by up to 50%. This research enhances the enzymatic precision for TBL biosynthesis and offers a robust foundation for its industrial-scale production, with broader implications for the engineering and in silico analysis of glycosyltransferases.

## 1. Introduction

Trilobatin (TBL) is a dihydrochalcone sweetener from the Chinese sweet tea plant (*Rubus suavissimus S. Lee*) [1] and is classified as a flavonoid. Recently, diseases such as obesity and diabetes caused by excessive intake of traditional sugars such as sucrose have severely threatened human health [2,3]. As the contradiction between the human desire for sweet taste and the demand for a healthier lifestyle has increased, the market need for low-calorie sweeteners has become substantially higher [4]. Being an extremely low-calorie natural sweetener, TBL has a sweetness 300 times higher than that of sucrose [5,6] and is thus a highly promising sugar substitute.

Currently, TBL is primarily produced using the Chinese sweet tea plant extract [7,8], with a heavy reliance on the leaves of this plant. This extraction largely depends on specific harvesting times of the leaves, which greatly limits the industrial demand for TBL raw materials. Additionally, different dihydrochalcones have varying heat sensitivities and are transformed or degraded during thermal processing, resulting in significant TBL loss and lower extraction efficiency during extraction [9,10]. By contrast, biosynthesis is not limited by time, space, or material supply. Because biosynthesis is conducted under mild conditions and has a high theoretical conversion rate [11,12,13], it is more suitable for mass industrial production. With the advancement in sequencing technology, an increasing number of genes have been discovered in plants, thereby making TBL biosynthesis feasible. In 2017, Eichenberger et al. synthesized a series of dihydrochalcones, including TBL, using baker’s yeast [14]. By introducing various heterologous proteins such as 4CL, CHS, and DBR, they reconfigured the yeast’s flavonoid metabolic pathway. They successfully synthesized TBL from scratch in the yeast chassis (yield: 36 mg/L), which proved the viability of TBL biosynthesis. Nevertheless, the broad substrate specificity of the glycosyltransferase used generally resulted in low dihydrochalcone production. Yahyaa et al. identified a UDPG glycosyltransferase, phloretin-4′-O-glycosyltransferase, from *Malus x domestica Borkh* (*Md*ph-4′-OGT) that efficiently converts phloretin into TBL [15]. A TBL yield of 107 mg/L was achieved using *Escherichia coli* to express this enzyme, which resulted in a whole-cell catalytic reaction from phloretin to TBL. In 2020, Wang et al. identified another UDPG glycosyltransferase, PGT2, that catalyzes TBL synthesis from phloretin in apples [16]. On overexpressing this gene in apple and tobacco leaves, they could increase the TBL yield to 100 mg/g DW. Although these biological TBL synthesis studies have provided new ways of synthesizing TBL, the reported TBL yields remained relatively low. This is partly because of the shortage and low activity of key enzymes in TBL synthesis and because of by-product generation during synthesis. Both of these factors limit the increase in biosynthetic production and large-scale application of TBL [17,18]. Therefore, unearthing new key enzymes in TBL synthesis, enhancing enzyme activity and specificity, and reducing by-product yield may provide new ideas for increasing TBL yields through biosynthesis.

In our previous studies, we optimized the biosynthesis of the precursor of TBL biosynthesis [19], phloretin, and thus resolved the issue of the TBL precursor supply. In this study, by using the *Md*ph-4′-OGT sequence as a probe for ph-4′-OGTgene mining, a novel key enzyme was obtained in TBL synthesis. Through optimizations such as reaction condition fine-tuning and mutant screening, we increased TBL production and the key enzyme’s specificity, whereas we reduced the by-product yield. These findings offer new insights and strategies for improving TBL biosynthesis.

## 2. Results and Discussion

### 2.1. Screening of ph-4′-OGT

**Figure 1:**
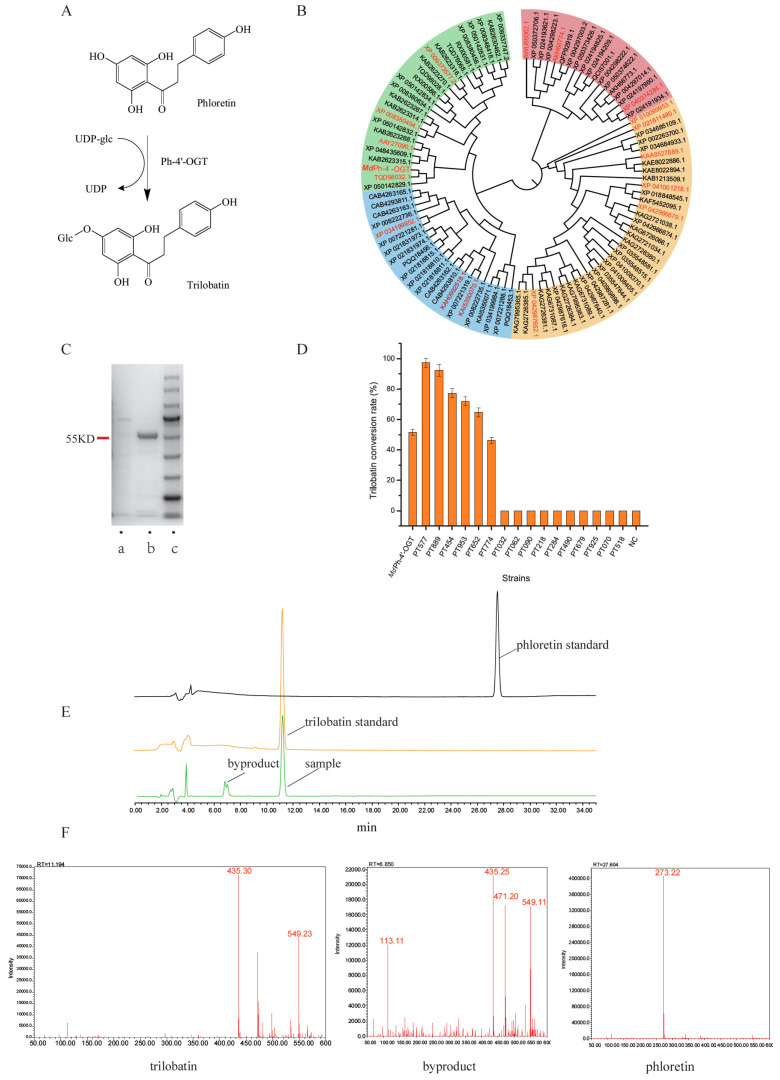
The screening of ph-4′-OGT. (**A**) Catalytic synthesis of TBL using phloretin as the substrate; (**B**) Construction of a gene tree. The green region represents species from the apple genus, the blue region denotes species from the apricot genus, and the red region signifies species from the strawberry genus. All these species belong to the Rosaceae family. The yellow region encompasses various species from the Moraceae, Euphorbiaceae, Vitaceae, Myricaceae, and Juglandaceae families. The ph-4-ogt in red fonts designates the selected glycosyltransferase; (**C**) The expression of ph-4′OGT proteins. ‘a’ stands for the empty plasmid control, ‘b’ stands for the ph-4′-OGT protein, and ‘c’ represents the marker. The position indicated by the red line approximately corresponds to the ph-4′-OGT with a size of ~55 kDa; (**D**) Screening of different ph-4′-OGT catalytic activities; (**E**) HPLC for TBL, phloretin, and sample. The elution times for phloretin and TBL are 27.8 min and 11.2 min, respectively, and a by-product is observed at around 6.9 min. (**F**) Mass spectrometry for TBL, phloretin, and byproduct. The characteristic peaks of trilobatin and phloretin are 435 and 273, respectively. The mass spectrometry information for the sample and the standard are consistent. The spectral data for the byproduct are similar to that of trilobatin, suggesting that it may be an isomer of trilobatin, possibly phloridzin.

During experimental verification, *Md*ph-4′-OGT exhibited strong phloretin glycosyltransferase activity, which facilitated the catalytic reaction of phloretin and the glycosyl donor UDPG to generate TBL (Figure 1A). However, the TBL conversion yield with *Md*ph-4′-OGT was only approximately 53%. This amount is not equivalent to the amount of TBL expected to be produced through large-scale industrial production. Hence, gene mining and the acquisition of a more efficient enzyme are crucial for producing TBL at the industrial scale. *Md*ph-4′-OGT is a glycosyl transferase derived from *M. x domestica Borkh* and exhibits the most extensively reported ph-4′-OGT functionality thus far [15]. Using this sequence as a probe, 100 homologous protein sequences were obtained through their alignment with sequences available in the NCBI database, and this formed the initial ph-4′-OGT screening sequence. A gene tree was constructed using MEGA software (https://github.com/meganz/MEGAsync, accessed on 2 December 2023) [20].

In the constructed gene tree, the 100 sequences demonstrated an evolutionary divergence into four clades. Based on evolutionary proximities and categorical groupings, 17 sequences were judiciously selected and predicted to exhibit phloretin glycosyltransferase activity. These 17 sequences included the sequence for the previously characterized *Md*ph-4′-OGT (Figure 1B).

The 17 selected phloretin glycosyltransferase gene sequences were synthesized by Wuhan Genecreat Bioengineering Co., Ltd. (Wuhan, China), who employed strategic codon optimization for *E. coli* expression. By applying the Gibson assembly protocol [21,22], these genes were integrated into the pET-28a expression vector.

All sequences on the pET-28a vector were transformed into *E. coli* BL21 (DE3). Colony PCR was performed on picked colonies to confirm the successful transformation. The validated colonies were subsequently cultured in 2YT medium at 37 °C and 220 rpm. When the OD600 reached approximately 0.8, the culture temperature was reduced to 16 °C, and 0.5 mM IPTG was added to induce target protein expression. Cultivation was sustained for an additional 14–16 h, and the cells were then harvested and lysed using a high-pressure homogenizer operating at 900 bar for 5 min.

Following centrifugation at 4 °C and 5000 rpm for 1 h, the clear supernatant was retrieved, and the protein constituents of the cells were purified through nickel-affinity chromatography. The purity and molecular mass of the isolated proteins were verified through SDS-PAGE, and it was evident that the molecular weight of the target protein was approximately 55 kDa (Figure 1C). Thereafter, the concentration of each purified protein was determined, and the proteins were duly introduced into their reaction systems to detect their activities. After 3 h of reaction at 37 °C, TBL and phloretin quantities in each reaction system were analyzed through high-performance liquid chromatography (HPLC).

The results of the 17 ph-4′-OGT sequences, including *Md*ph-4′-OGT, were evaluated (Figure 1D). Moreover, the product TBL and the substrate phloretin were identified through HPLC and mass spectrometry (MS; Figure 1E). Interestingly, the emergence of specific by-products was observed. The MS analysis supported the hypothesis that the by-product is a TBL isomer, specifically identified as phloridzin. It was also indicated that glycosyltransferases are capable of attaching glycosyl groups at the 2 and 4 positions of phloretin [8,16,23]. Among the 17 sequences, the average activity of PT577 was 95.3%, with a maximum conversion yield of 97.7%. Thus, PT577 showcased the highest ph-4′-OGT activity across all sequences. Other sequences PT889, PT454, PT953, and PT652 also displayed good ph-4′-OGT activities, and their conversion yields were 92.0%, 74.1%, 70.7%, and 66.1%, respectively, all surpassing the catalytic capacity of *Md*ph-4′-OGT. PT774 had a conversion yield of 46.1%, slightly lower than that of *Md*ph-4′-OGT. The remaining seven sequences produced no TBL in repeated experiments, which indicated they lacked the expected ph-4′-OGT activity.

Overall, through experimental screening, the activities of all 17 predicted ph-4′-OGT sequences were evaluated. Six sequences with ph-4′-OGT activity, namely PT577, PT889, PT454, PT953, PT652, and PT774, were obtained. PT577, isolated from the species *P. x bretschneideri* (Chinese white pear) and logged in the NCBI as XP_009373577.2, is considered the best ph-4′-OGT identified and its TBL conversion yield reaches up to 97% ± 3%.

### 2.2. Optimization of PT577 Glycosyltransferase

**Figure 2:**
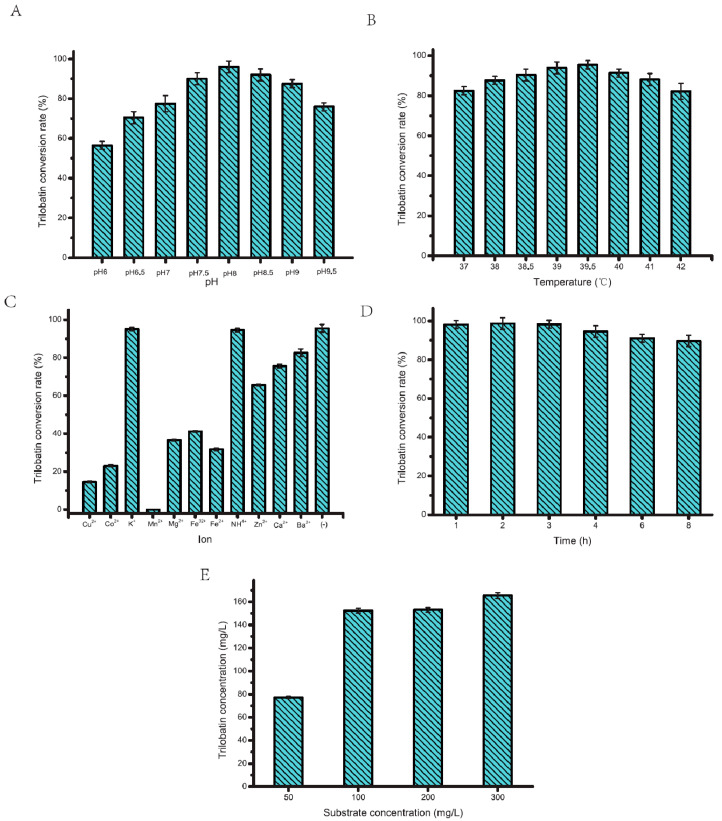
Catalytic reaction parameters optimization for PT577 glycosyltransferase. (**A**) Impact of pH for PT577; (**B**) Effect of temperature on the PT577 activity; (**C**) Metal ions test for TBL production of PT577; (**D**) Time optimization for PT577 reactions; and (**E**) Relationship between substrate concentration and TBL yields as to PT577.

PT577 is an efficient ph-4′-OGT obtained through gene mining. The reaction conditions of this enzyme, including pH, reaction temperature, metal ion, reaction time, and substrate concentration, were optimized to further improve its catalytic activity. The pH of the enzyme reaction system is a crucial factor for enzymatic transformations in biosynthesis, as it can impact the reaction by altering the substrate’s dissociation status and influencing the substrate’s interaction with the enzyme’s binding sites [24,25]. To study the effect of the reaction system pH on the PT577catalytic efficiency, pH buffer solutions with varying pHs (pH 6, pH 6.5, pH 7.0, pH 7.5, pH 8, pH 8.5, pH 9, and pH 9.5) were prepared for reaction systems at 37 °C, and the TBL conversion yield obtained through the reaction of PT577 under these pH gradients for 2 h was measured (Figure 2A). According to the experimental data, extremely acidic (pH < 7) or alkaline (pH > 9) conditions markedly diminished the catalytic efficiency of the enzyme studied. pH may exert a multifaceted influence on enzyme catalysis. One possible reason is that pH fluctuations can alter the ionization states of pivotal amino acid residues at the enzyme’s active site, catalytic groups, and ionization necessary for proton donors or acceptors. Additionally, pH may modulate substrate dissociation in the solution, thereby affecting the enzyme–substrate interaction dynamics. Furthermore, changes in pH can alter the enzyme’s tertiary structure within the solution, thus influencing the relative spatial positioning between the substrate and the enzyme’s catalytic center during binding, which then influences the biochemical reaction. The enzyme exhibits optimal activity at pH 8.0 and retains >90% of its catalytic conversion yield between pH 7.5 and 9.0. Thus, the enzyme has a degree of adaptability and may be appropriate for scaling up the industrial production of TBL.

Ambient temperature significantly affects the demonstrated catalytic activity by inducing changes in the three-dimensional (3D) configuration of protein molecules [26,27], morphology of substrate-binding sites, and architecture of the enzyme’s active site. The reaction temperature influences enzyme catalysis in a dualistic manner. On the one hand, elevated temperatures enhance molecular kinetics, thereby increasing the chances of enzyme–substrate collisions and correspondingly increasing enzyme activity. On the other hand, excessive thermal energy can cause enzyme protein denaturation, thereby disrupting its native conformation and inducing a precipitous drop in its catalytic function. Studying PT577′s catalytic activity at different temperatures and determining the optimal temperature for exerting its catalytic action are of great significance. So, in a pH 8.0 environment, eight different temperature gradients, namely 37.0 °C, 38.0 °C, 38.5 °C, 39.0 °C, 39.5 °C, 40.0 °C, 41.0 °C, and 42.0 °C were used to conduct in vitro enzymatic reactions. When the temperature reached 38 °C, the catalytic activity of PT577 significantly increased and reached its peak at 39.5 °C (Figure 2B). This temperature was considered the optimal reaction temperature for PT577. When the temperature dropped to between 39 °C and 40 °C, PT577 retained a high catalytic activity. This temperature range is relatively easy to control for industrial production, which re-asserts the potential of PT577 for industrial production.

Metal ions frequently serve as essential cofactors in enzymatic catalysis, often increasing enzyme activity within certain biochemical reactions [28,29]. As “conductors” in enzymatic processes, these ions facilitate electron transfer and forge connections between enzyme active sites and substrates. They may also interact with amino acid residues on the enzyme to strengthen the enzyme’s structural integrity or modify its spatial arrangement, consequently augmenting the catalytic efficiency of the enzyme. Typically, these metal ions are selective and exert their effects predominantly on metal-dependent enzymes. The experimental results demonstrate that almost all metal ions interfere with PT577′s catalytic activity (Figure 2C). More specifically, heavy metal ions with higher charges, such as Cu^2+^, Co^2+^, Mn^2+^, Fe^3+^, and Fe^2+^, tend to more severely affect PT577, whereas common light metal ions such as K^+^ and NH4^+^ have less of an impact.

PT577-mediated catalysis of phloretin and the glycosyl donor UDPG to form TBL requires a certain reaction time. Considering economic efficiency and time costs, the optimal reaction time of the enzyme must be explored [30,31]. The yield rate of PT577-catalyzed TBL conversion at different times (1, 2, 3, 4, 6, and 8 h) was calculated to determine an optimal reaction time. Within the initial 3-h period of the reactions, the rate of PT577-catalyzed TBL production in the assay was high, ranging from 95% to 97% (Figure 2D). However, the TBL yield decreased when the reaction was extended beyond 4 h. This decrease is postulated to occur due to prolonged reaction times that induce non-specific PT577 activities, thereby promoting by-product formation. Additionally, at elevated temperatures, the intrinsically potent antioxidant, TBL, may undergo certain degradation. These factors, individually or combined, likely contribute to the observed reduction in the TBL production rate with an increase in the reaction duration. Consequently, for pure PT577-utilizing enzymatic reactions, the optimal timeframe for achieving the maximum TBL conversion yield was concluded to be 3 h.

By increasing the UDPG content in the reaction system and increasing the phloretin substrate concentration [32,33], TBL production using PT577 may be increased. Phloretin is highly soluble in the eco-friendly solvent butanediol and dissolves at a 67.2 g/L concentration at ambient temperature. To facilitate a high-throughput conversion into phloretin, an expansive reaction system was established for processing phloretin substrate concentrations of 50, 100, 200, and 300 mg/L. This system also incorporated 3 mM of UDPG, 50 mM Tris-HCl buffer at pH 8.0, and 0.1 mg/mL of the enzyme. According to the results (Figure 2E), upon elevating the initial phloretin concentration to 100 mg/L within the system, the TBL conversion yield corresponding to the supplemental quantities of the phloretin substrate increased notably. The total conversion efficiency of the system was consistently >95%. However, beyond this initial phloretin concentration, incremental additions did not significantly increase the TBL conversion yield; instead, the overall conversion efficiency to TBL was considerably reduced. The experimental data also revealed that the maximum TBL yield was 163.3 mg/L at an initial phloretin concentration of 300 mg/L. This was the most substantial yield recorded to date, which marked a 52% improvement over the highest TBL yields reported earlier.

In summary, the optimal pH and temperature for PT577′s enzymatic reaction are 8 and 39.5 °C, respectively. This enzyme displayed the highest reaction activity in the absence of external metal ions. Interference from metal ions rather impairs normal catalytic activity. The optimal reaction time was within 3 h, and longer reaction times led to a decline in TBL yield. When the substrate concentration was 100 mg/L, PT577 achieved the optimal catalytic activity with high selectivity. Moreover, the current study achieved a TBL biological yield of 163.3 mg/L, the highest yield reported to date.

### 2.3. Modification to Enhance Activity and Specificity of PT577

**Figure 3:**
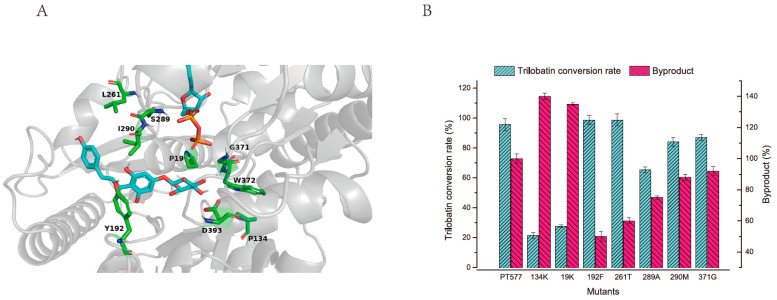
Optimization of activity and specificity of PT577. (**A**) Simulation results of PT577 molecular structure. The grey structures represent the beta-sheets or alpha-helices, the green residues are closely related to trilobatin and may affect the activity of PT577, and the blue structures are those of trilobatin and the sugar donor, respectively; (**B**) Mutant activity testing for TBL and by-product.

To enhance PT577 specificity and reduce byproduct production, we conducted structural engineering and optimization of the enzyme. The precise 3D structural information of the enzyme was first acquired to enable targeted selection of active sites for modification. Such strategic modifications augment specificity and catalytic efficiency. For this endeavor, the AlphaFold2 deep learning algorithm was used to predict the PT577 3D structure [34]. Based on this foundation, the structural prediction module of AlphaFold2 computed the position and interatomic distances of all amino acids, the geometry of the peptide bonds, including bond lengths and angles, and torsion angles within the amino acid residues (Figure 3A). Thus, 3D coordinates for all heavy atoms in the glycosyltransferase PT577 were accurately predicted, thereby providing a robust framework for precise enzyme engineering.

To reduce the generation of the by-product phloridzin, this study increased the binding energies of the reaction product TBL and the enzyme core site [35,36]. When the binding energies of TBL and enzyme increased, phloretin reacted to produce TBL instead of the by-product [37]. After the substrate and enzyme were bound, they were both deformed, entered a transition state, and lowered the reaction activation energy, thus increasing the reaction rate. Binding free energy is a critical index for characterizing the strength of enzyme’s binding to the substrate. It can qualitatively analyze the ability of the enzyme to bind to different substrates. Analyzing and mutating these hot spot residues are more advantageous for accurately studying the interaction between the enzyme and small molecule substrates and provide guidance for the rational design of enzymes. To navigate as many amino acid residue sites around TBL as possible and reduce the experiment volume, a computer virtual mutation method was implemented for the residues in the enzyme-binding pocket.

Rosetta [38,39] was used to dock ph-4′-OGT and TBL molecules. Based on the docking results, their binding methods and related amino acid residue sites were analyzed (Figure 3A). Generally, the amino acid residues constituting the substrate-binding pocket significantly affect enzyme activity. Therefore, the substrate binding-related sites can be mutated to increase the enzyme’s affinity for the substrate.

Virtual mutation was finally calculated by selecting the lowest binding energy amino acid calculation results from each mutation site. Eventually, the optimum virtual mutation results of seven site residues, namely 134K, 19K, 192F, 261T, 289A, 290M, and 371G, were obtained. Following experimental confirmation and employing a 3-h reaction sample to determine the yield of TBL, the results demonstrated a relative increase in the conversion efficiency of the TBL by the mutants, alongside a decrease in the formation of by-products (Figure 3B). In the assay results of the two mutants, 192F and 261T, the TBL conversion yield exceeded 99%, signifying an improvement over the parent enzyme PT577. Additionally, these targeted site mutations effectively suppressed the by-product activity linked with PT577, with the 192F and 261T mutations achieving 50% and 40% reductions, respectively.

## 3. Materials and Methods

### 3.1. Materials

Competent *E. coli* BL21 (DE3) cells were obtained from Beijing TransGen Biotech Co., Ltd. (Beijing, China). Phloretin, TBL, and UDP-glucose were purchased from Beijing Solarbio Science & Technology Co., Ltd. (Beijing, China).

### 3.2. Construction of Recombinant Plasmid and Ph-4′-OGT Gene Mining

Using the *Md*ph-4′-OGT protein sequence (Uniprot No.: A7MAS5) as a probe, a homologous sequence was searched for by using the psi-blast function of the BLASTP program of NCBI. The top 100 sequences were selected based on confidence levels to construct a phylogenetic tree. Seventeen sequences were selected from different branches of the gene tree for further screening. The exact experimental procedure was as follows. Sequences in the top 100 based on confidence were aligned through multiple sequence alignment. After alignment, the sequences were refined by removing gaps using Gblocks [40,41], followed by the construction of the gene tree by using MEGA software (https://github.com/meganz/MEGAsync, accessed on 2 December 2023) employing the neighbor-joining method [42,43]. Default settings were applied for the model and bootstrap parameters to generate the gene tree. The 17 selected sequences (Table S1) underwent codon optimization and were synthesized by Wuhan GeneCreate Biological Engineering Co., Ltd. (Wuhan, China). During synthesis, a 6× histidine (His) tag at the C-terminus of the target gene and an LE flexible amino acid linker were incorporated to prevent any adverse effects on the protein function. Fragments of the desired gene and pET28a vector were amplified and cloned using a PCR and fragment ligation system to construct the recombinant plasmid. Gene synthesis and plasmid assembly were performed by Wuhan GeneCreate Biological Engineering Co., Ltd. (Wuhan, China).

### 3.3. Expression of ph-4′-OGT

A 5 μL volume of the *E. coli* glycerol stock was inoculated into 5 mL LB broth supplemented with kanamycin to achieve 100 μg/mL, and the culture was incubated overnight at 37 °C with agitation at 220 rpm. Subsequently, a 1% volume of this pre-culture was transferred to a 2 L Erlenmeyer flask containing 500 mL of 2× YT medium supplemented with kanamycin at 100 μg/mL. This culture was then incubated at 37 °C with shaking at 220 rpm for 4–6 h until an OD600 of 0.6–0.8 was attained. The culture temperature was then reduced to 16 °C before the culture was induced with 0.5 mM IPTG. Following induction, the culture was incubated at 16 °C and 220 rpm for 16–20 h to promote overexpression of the target recombinant enzyme. Subsequently, the induced cells were centrifuged using large-capacity tubes, precisely balanced to under 0.1 g discrepancy, at 6000 rpm for 20 min at 4 °C. The supernatant was discarded, and the cell pellet was washed through resuspension in 40 mL of a pH 8.0 buffer containing 50 mM Tris-HCl and 10% glycerol. This suspension was centrifuged at 8000 rpm for 10 min at 4 °C. After the wash supernatant was discarded, the cell pellet was collected and centrifuged again to ensure complete cleansing. The final washed pellet was resuspended in 50 mL of the same protein buffer and lysed using a high-pressure homogenizer at a controlled temperature of 4 °C. Optimal lysis of *E. coli* cells was achieved between 800 and 900 bar with a total lysis duration of 5 min. The cell lysate was centrifuged at 8000 rpm for 1 h at 4 °C, and the resultant clear supernatant was harvested as the crude extract containing the enzyme of interest.

### 3.4. Protein Purification of ph-4′-OGT Enzyme

Proteins were purified based on the principles of nickel ion affinity chromatography. The target protein was tagged with a 6× His tag and so could bind to the Ni^2+^-embedded agarose gel beads, which allowed for its separation from impurities and subsequent elution with imidazole solutions to obtain a purified form. Some contaminant proteins also exhibited affinity for nickel ions; therefore, the target protein could not be directly purified. Hence, a gradient of imidazole solutions was used to determine the elution concentration that could effectively separate the target protein from impurities. To establish the optimal conditions for protein purification, several experimental imidazole gradient elutions were performed. To prevent the deactivation of the recombinant enzyme caused by high temperatures, all separation and purification steps were carried out at low temperatures. The specific steps were as follows. The nickel column was rinsed twice with double the column volume of H_2_O and equilibrated with double the column volume of 10 mM imidazole protein buffer. The crude enzyme extract was slowly applied to the gravity column and allowed to pass through the nickel resin. The flow-through was collected, and the process was repeated. Subsequently, the sample that passed through the column was collected. The column was washed twice with double the column volume of protein buffer. Then, the gradient elution was performed using 30 mL of 20, 50, 100, 200, and finally 300 mM imidazole protein buffer, respectively. Samples from the initial drops of each elution fraction were collected for further analysis. SDS-PAGE was used as a guide to ultrafiltrate the eluted target protein. Ultrafiltration devices with a molecular weight cutoff that is one-third smaller than the target protein were thus selected. The solution in the tube was concentrated to approximately 1 mL. After this, protein buffer was added to attain a total volume of 5 mL and centrifugation was continued, thereby concentrating the solution to approximately 500 µL at 4 °C and 5000 rpm for about 30 min. This process was repeated 2–3 times. A small portion of the solution was used for SDS-PAGE electrophoresis analysis and concentration determination, whereas the remaining sample was flash-frozen in liquid nitrogen and stored at −80 °C.

### 3.5. Enzymatic Reaction with Ph-4′-OGT Enzyme

A 200-μL reaction system containing 2 mM UDPG, 50 µM phloretin, and 0.1 mg/L of the purified enzyme in a 50 mM Tris-HCl buffer at pH 8.0 was prepared. The respective reaction components were added to a 1.5-mL centrifuge tube, and the total volume was topped up to 0.2 mL with ultrapure water. The reaction mixture was incubated for 3 h at 37 °C and 220 rpm in a temperature-controlled orbital shaker, and the reaction was terminated by adding 20 µL of trichloroacetic acid solution.

### 3.6. Detection of Reaction Products

Mass spectrometry was performed with a Waters Alliance HPLC e2695 system (Sunnyvale, CA, USA). An Inertsil^TM^ ODS-3 C18 column was used to perform reversed-phase chromatography. The mobile phase comprised acetonitrile with 0.1% formic acid and ultrapure water with 0.1% formic acid. The mobile phase was filtered through a 0.22-μm organic/water system membrane to remove impurities and degassed through ultrasonication for at least 20 min before use. The residual phloretin and the amount of TBL produced in the reaction system were measured through HPLC. The target analytes exhibited UV absorption peaks at 280 nm, which was the detection wavelength of choice. The chromatographic column used was a Shodex C18-120-5 (Shenyang, China) silica gel-based reversed-phase column (Showa Denko K.K., Japan). The column temperature maintained was 40 °C (within a ±2 °C range), and the injection volume was set at 10 µL. For the HPLC gradient elution program (Table S2), channel A comprised acetonitrile with 0.1% formic acid, and channel B consisted of ultrapure water with 0.1% formic acid. Then, to 100 µL of the reaction supernatant, an equal volume of methanol was added, and the mixture was extracted through ultrasonication for 30 min. The mixture was centrifuged at 8000 rpm for 10 min. The clear supernatant was used for HPLC analysis. Concentrations of substrates and products within the sample were calculated based on their specific peak areas at 280 nm by plotting a calibration curve for determination.

### 3.7. Optimization of PT577 Glycosyltransferase

PT577 is an engineered sequence exhibiting high ph-4′-OGT activity and was identified through two iterative rounds of genomic screening. A univariate analysis was performed to delineate the characteristic performance of the enzyme under varying reaction conditions. To establish the optimal pH for the PT577 enzymatic activity, a series of reactions were initiated in Tris-HCl buffers and phosphate buffers (PBS) having pH values adjusted from 6.5 to 9.0. The concentrations of generated products were quantified to allow enzyme yield calculation. The most propitious pH for catalysis was adjudicated by comparing substrate conversion efficiencies across the pH spectrum. Thermal optimization of the enzyme activity was determined by executing reactions over 37.0 °C–42.0 °C. The post-reaction analysis involved measuring product concentrations and calculating resultant yields. The peak temperature favorable for substrate conversion was established on the basis of the results of comparative yield evaluation. To identify the metal ion that best supports the catalytic function of PT577, an array of divalent cations, specifically Ca^2+^, Co^2+^, Cu^2+^, Fe^2+^, Fe^3+^, Mg^2+^, Mn^2+^, Ni^2+^, and Zn^2+^, were each incorporated at 5 mM into the reaction milieu. Deionized water was used as a baseline control in the absence of metal ions. The substrate turnover efficiency was assessed under each ionic condition to identify the metal ion most favorable for catalysis. The impact of the substrate concentration on the TBL yield of the enzyme was thoroughly investigated by using four concentrations of the substrate: 50, 100, 200, and 300 mg/L. These concentrations were methodically selected to evaluate the relationship between substrate availability and enzyme-catalyzed TBL production. Finally, to determine the ideal reaction duration for maximum catalytic turnover, enzyme-mediated reactions were traversed across a temporal gradient extending from 1 to 12 h. Product concentrations were monitored, and enzyme yields were computed. This facilitated the identification of the optimal catalysis timeframe derived from efficiency comparisons across varying durations.

### 3.8. Modification of PT577

After a high-yielding PT577 enzyme variant was isolated through iterative rounds of experimental selection, we aimed to further refine its function. First, the advanced predictive capabilities of AlphaFold2 were employed to infer its three-dimensional conformation. The ensemble of possible structures was evaluated. The variant exhibiting the highest predicted Local Distance Difference Test (pLDDT) score was selected as our template for subsequent modifications [44,45]. For the molecular interaction analysis, the ligand docking module within the Rosetta suite was engaged to model PT577 binding with the ligands TBL and UDPG. The UDPG structure was retrieved from the ZINC database (https://zinc.docking.org/, accessed on 4 December 2023), and the TBL configuration was designed using Schrödinger’s suite of computational tools. Exhaustive conformational analysis on the ligand structures was performed using Schrödinger’s search algorithms, with the outputs formatted in sdf files. These files were then converted into parameter files compatible with Rosetta’s docking protocols. The AlphaFold2-generated theoretical model was further refined using Schrödinger’s protein preparation tools, which accounted for charge distributions under experimental pH conditions and facilitated energy minimization. Using Rosetta’s Cartesian_ddG method, virtual mutagenesis was explored to quantify the thermodynamic implications of amino acid substitutions. Structural optimization in this study proceeded in two phases. Initially, the optimal side-chain positions for native and mutant amino acid residues were determined. Thereafter, a comprehensive relaxation of the enzyme–ligand complex, focusing on residues within a 6 Å TBL radius, was performed to achieve a minimum energy conformation. After this, comparative energy assessments before and after the mutation indicated favorable candidate mutations where the binding energy differential was negative. In accordance with our understanding of the enzyme’s catalytic ability, experimentally directed mutagenesis was targeted to these computational hotspots. Each potential mutation site underwent a rigorous in silico saturation mutagenesis, and the primers used were presented in Table S3. The delta in binding free energy consequent to each substitution was calculated. The detailed calculative nuances are meticulously documented in Table S4.

## 4. Conclusions

The main objective of this project is to screen the key enzyme, phloretin 4-O-glycosyltransferase, in TBL biosynthesis to augment the yield of TBL through biological synthesis. The poor reaction specificity of ph-4′-OGT was improved through modification under the guidance of computational virtual mutation. Using the *Md*ph-4′-OGT protein sequence as the probe and relying on the non-redundant protein sequence database of NCBI, 100 homologous sequences of the *Md*ph-4′-OGT protein were obtained. Evolutionary analysis was performed with the set of homologous sequences, a gene tree was built, and 17 sequences expected to have phloretin glycosyltransferase activity were screened. This screening was based on the location of the probe sequence and other factors such as species origin and evolutionary distance. The activities of all the 17 predicted ph-4′-OGT sequences were experimentally verified, and an additional six sequences with ph-4′-OGT activity, namely PT577, PT889, PT454, PT953, PT652, and PT774, were ultimately obtained. Among these, the PT577 enzyme produced a TBL yield of 95% ± 3%. Therefore, we considered it to be the optimal ph-4′-OGT enzyme among the other enzymes screened. This enzyme was sourced from the Chinese white pear *P. x bretschneideri* species with the NCBI accession number as XP_009373577.2. The optimal reaction conditions of the PT577 enzyme were determined through single-variable analysis. The optimal pH of the PT577′s reaction was 8, and the optimal temperature was 39.5 °C. The highest reaction activity of this enzyme required no external metal ions. Metal ion introduction would, in turn, disrupt the normal catalytic activity of PT577. PT577 exhibited optimal catalytic activity when the substrate concentration was 100 mg/L. The optimal reaction time, as determined, was within 3 h. The highest reported yield of TBL through biosynthesis was 163.3 mg/L, which is a 52% boost to the highest yield reported earlier. PT577 and TBL molecules were docked using Rosetta. All amino acids within a 5 Å distance from the TBL molecule were selected as to-be-mutated amino acids, and virtual saturation mutation was conducted on these amino acids. Based on the change in binding energy, we selected mutants exhibiting stronger affinity to TBL and eventually obtained seven optimum mutation amino acid sites. Function validation of these mutants was performed, and two mutant proteins, PT577-192F, and PT577-261T, were acquired. The catalytic activities of these mutant proteins were further elevated in comparison with that of PT577, thereby achieving a high TBL yield of 99.2%. The by-product generation potential was reduced by 50% and 40%, respectively. Transfer of the PT577 enzyme into phloretin-producing yeast could be attempted to extend the path of biological production of TBL and reduce production costs. The sugar donor used in the catalytic reaction system was UDPG, but it is expensive and not conducive to large-scale industrial applications. We need to explore cheaper sugar donors further, increase endogenous UDPG supply in *E. coli* through metabolic engineering modification, or directly achieve UDPG recycling in the reaction system.

## Data Availability

The raw data that support this study are available from the corresponding authors (Xianqing Chen and Xiao Wang) upon reasonable request.

## Supplementary Materials

The following supporting information can be downloaded at: https://www.mdpi.com/article/10.3390/molecules29030643/s1, Table S1. Gene List for ph-4′-OGTs; Table S2. Gradient elution procedure of HPLC for trilobatin; Table S3. Primers for PT577.2 half-saturated site mutations; Table S4. Virtual mutation by PT577.
