# Supplementary material for: Optimizing Trilobatin Production via Screening and Modification of Glycosyltransferases
Source: Molecules. 2024 Jan 30;29(3):643. doi: 10.3390/molecules29030643 (PMC10856287; doi:10.3390/molecules29030643)
Supplement: Supplementary file 1 [file molecules-29-00643-s001.zip › molecules-2816240-supplementary.pdf]

Optimizing trilobatin production via screening and modification of Glycosyltransferases

Yue Yang [1,2,†], Yuhan Cheng [2,†], Tao Bai [1,†], Shimeng Liu [1], Qiuhui Du [1], Wenhao Xia [2], Yi Liu [2], Xiao Wang [1,*] and Xianqing Chen [1,*]

Supporting information

Table S1. Genes list for this study.

Table S2. Gradient elution procedure of HPLC for trilobatin.

Table S3. Primers for PT577.2 half-saturated site mutations.

Table S4. Virtual mutation for PT577.

Table S1. Genes list for this study

No.	Organisms	NCBI No.
PT032	*Malus baccata*	TQD98032.1
PT062	*Rubus chingii var. suavissimus*	AWU66062.1
PT090	*Pyrus communis*	AAY27090.1
PT218	*Juglans microcarpa x Juglans regia*	XP_041001218.1
PT284	*Rosa chinensis*	XP_040374284.1
PT454	*Malus domestica*	XP_008380454.1
PT490	*Manihot esculenta*	XP_021611490.1
PT577	*Pyrus x bretschneideri*	XP_009373577.2
PT652	*Carya illinoinensis*	XP_042987652.1s
PT679	*Carya illinoinensis*	XP_042966679.1
PT774	*Fragaria x ananassa*	AKH66774.1
PT889	*Nyssa sinensis*	KAA8527889.1
PT953	*Morus notabilis*	XP_010099953.1
PT925	*Prunus dulcis*	XP 034199952.1
PT070	*Prunus dulcis*	KAI5350070.1
PT518	*Plasmodium falciparum*	CAA68280.1

Table S2. Gradient elution procedure of HPLC for trilobatin

Time (min)	Flow rate (mL/min)	A%	B%	C%	D%
0	0.8	28	72	0	0
14	0.8	28	72	0	0
30	0.8	40	60	0	0
35	0.8	28	72	0	0

Table S3. Primers for PT577.2 half-saturated site mutations

Primers	Sequences
11F-TB	atggtgcagcatcgttttctggttctgaccnbr
11F-r	ggtcagaaccagaaaacgatgctgcaccat
112L-TB	tcatccgtatacctgcctggtgtataccattnbs
112L-r	aatggtatacaccaggcaggtatacggatga
132I-TB	tgaactgcatctgccgaatgttctgctgtggnbs
132I-r	ccacagcagaacattcggcagatgcagttca
373H-r	ccagccgcaatgggtcacaaagcagcccag
373H-TB	ctgggctgctttgtgacccattgcggctggmrv
12L-r	aaaggtcagaaccagaaaacgatgctgcac
12L-TB	gtgcagcatcgttttctggttctgacctttnbs
133I-r	aatccacagcagaacattcggcagatgcag
133I-TB	ctgcatctgccgaatgttctgctgtggattnbs
13V-r	cggaaaggtcagaaccagaaaacgatgctg
13V-TB	cagcatcgttttctggttctgacctttccgnbs
14A-r	tgccggaaaggtcagaaccagaaaacgatg
14A-TB	catcgttttctggttctgacctttccggcanbs
194I-r	actatacggattggtatccaccataaaact
194I-TB	agttttatggtggataccaatccgtatagtnbs
199F-r	cagcggcagtgcaaaactatacggattggt
199F-TB	accaatccgtatagttttgcactgccgctgnbs
261T-r	atcaccaccaaaacttttatccagcggaac
261T-TB	gttccgctggataaaagttttggtggtgatnvb
289A-r	gccaaaactcacataaatcacgctgccttc
289A-TB	gaaggcagcgtgatttatgtgagttttggcnbs
370A-r	atgggtcacaaagcagcccagactcgggct
370A-TB	agcccgagtctgggctgctttgtgacccatnbs
371G-r	gcaatgggtcacaaagcagcccagactcgg
371G-TB	ccgagtctgggctgctttgtgacccattgcnvb
Kana-f	aaaccgttattcattcgtgattgcgcctgagc
Kana-r	caggcgcaatcacgaatgaataacggtttg
12L-ST	gtgcagcatcgttttctggttctgacctttctc
13V-ST	cagcatcgttttctggttctgacctttccggta
14A-ST	catcgttttctggttctgacctttccggcagct
15P-ST	cgttttctggttctgacctttccggcacagcca
19K-ST	ctgacctttccggcacagggtcatattaataag
110D-ST	gaaggtcatccgtatacctgcctggtgtatgat
133I-ST	ctgcatctgccgaatgttctgctgtggattatc
134K-ST	catctgccgaatgttctgctgtggattcagaag
185H-ST	ctgaccagtcgtgatctgccgagtcat
192F-ST	ctgccgagttttatggtggataccaatccgttc
194I-ST	actatacggattggtatccaccataaaactatt

195L-ST	cgagttttatggtggataccaatccgtatagttttctc
261T-ST	gttccgctggataaaagttttggtggtgataca
289A-ST	gaaggcagcgtgatttatgtgagttttggcgca
290M-ST	ggcagcgtgatttatgtgagttttggcagtatg
291A-ST	agcgtgatttatgtgagttttggcagtattgca
369T-ST	agcagcccgagtctgggctgctttgtgaccaca
370A-ST	agcccgagtctgggctgctttgtgacccatgca
371G-ST	ccgagtctgggctgctttgtgacccattgcgga
372K-ST	gccgcaatgggtcacaaagcagcccagactaag

Table S4. Virtual mutation for PT577

Mutants sites	Binding energy	Mutants sites	Binding energy
110D	-6.8	132F	-4.18
110F	-1.46	132G	-0.4
110G	-5.73	132H	-1.28
110I	-2.45	132I	-8.01
110K	-0.22	132L	-4.41
110M	-0.74	132N	-1.82
110P	-2.28	132P	-0.14
110Q	-1.57	132Q	-2.39
110S	-1.25	132V	-1.26
110T	-0.75	133A	-2.18
110V	-4.48	133E	-1.05
11D	-1.28	133G	-2.59
11F	-6.18	133H	-3.1
11H	-0.14	133I	-4.41
11I	-2.72	133P	-0.77
11L	-0.3	133Q	-1.71
11M	-0.93	133R	-1.45
11T	-2.6	133S	-2.25
12C	-0.56	133V	-0.66
12F	-0.4	134E	-3.36
12K	-2.16	134F	-0.99
12L	-3.43	134G	-1.63
12Q	-2.14	134K	-6.12
12S	-0.83	13E	-0.22
12T	-0.5	13H	-1.04
12V	-0.58	13L	-1.95
12Y	-0.12	13P	-2.86
132A	-0.52	13S	-1.56
14P	-1.15	13T	-0.18
14Y	-2.37	13V	-3.42
15A	-2.75	14A	-4.53
15E	-0.66	14F	-0.21
15G	-1.34	14H	-1.44
15H	-4.8	14K	-0.44
15K	-0.14	14L	-3.32
15P	-5.62	16A	-0.94
15Q	-1.99	16G	-0.45
192K	-1.91	16K	-0.66
192M	-3.03	16P	-0.25
192S	-1.5	16S	-0.25
192V	-0.54	185E	-1.76
194A	-0.62	185H	-3.58

194C	-1.58	185T	-1.46
194I	-3.24	192F	-3.36
194M	-1	192H	-1.5
194N	-1.61	192I	-0.42
194P	-0.2	195N	-4.04
194Q	-0.39	195P	-0.86
194T	-1.06	195Q	-2.63
194Y	-1.67	195S	-3.54
195A	-0.07	195T	-0.83
195C	-0.06	195V	-2.36
195D	-1.2	199A	-1.13
195I	-0.87	199F	-3.25
195L	-5.25	199H	-0.44
199M	-0.22	199I	-0.88
199Y	-1.89	261C	-0.7
19A	-4.09	261E	-0.99
19D	-1.18	261F	-4.49
19F	-0.02	261G	-2.26
19G	-0.23	261H	-0.17
19H	-0.32	261M	-2.53
19K	-6.34	261N	-1.67
19L	-2.51	261Q	-1.39
19P	-0.14	261S	-3.01
19Y	-0.12	261T	-4.71
261A	-0.23	289A	-3.19
289T	-4.55	289C	-0.55
289V	-0.44	289D	-2.01
289Y	-1.32	289E	-1.55
290D	-1.94	289F	-0.42
290G	-2.3	289G	-3.96
290I	-0.65	289I	-0.68
290M	-5.99	289L	-3.02
290N	-2.34	289N	-1.79
290S	-4.02	289S	-0.11
289V	-0.44	290V	-2.64
289Y	-1.32	290Y	-5.96
290D	-1.94	291A	-4.52
290G	-2.3	291C	-0.26
290I	-0.65	291G	-1.91
290M	-5.99	291K	-1.66
290N	-2.34	291M	-1.56
290S	-4.02	291R	-2.36
290T	-0.04	291S	-2.16
369D	-4.22	291T	-1.85

369G	-0.89	291Y	-0.23
369I	-0.03	369A	-1.62
369L	-1.9	370A	-4.34
369M	-2.18	370C	-3.02
369N	-5.63	370G	-0.12
369P	-0.21	370H	-1.08
369S	-2.79	370I	-1.47
369T	-6.3	370L	-2.52
369V	-2.31	370M	-3.88
369Y	-0.07	370N	-0.53
372Q	-3.28	370S	-0.63
372S	-0.08	370V	-1.67
373D	-2.95	371D	-0.99
373H	-5.5	371G	-3.57
373I	-3.83	371N	-2.6
373N	-0.56	372A	-0.67
373R	-3.02	372F	-0.34
373V	-1.57	372G	-2.11
373Y	-0.34	372H	-0.19
372K	-5.71	372I	-2.03
